# DOES AUGMENTATION OF MITOCHONDRIAL THIOREDOXIN REDUCTASE 2 IMPROVE METABOLIC FITNESS?

**DOI:** 10.1093/geroni/igz038.1462

**Published:** 2019-11-08

**Authors:** Sandra Chocron, Andrew Pickering

**Affiliations:** 1 Barshop Institute for aging and longevity studies, University of Texas Health Science Center, San Antonio, Texas, United States; 2 Barshop Institute for aging and Longevity studies, UTHSCSA, San Antonio, Texas, United States

## Abstract

Mitochondrial Thioredoxin Reductase (TrxR2) is a rate limiting enzyme in the mitochondrial thioredoxin system which serves as one of the major mitochondrial ROS scavenging pathways. Txnrd2 is also a repressor of the ASK-1 oxidative stress induced apoptotic pathway. Our group previously identified a correlation with the expression of this protein and long-lived species and its overexpression prolonged lifespan in Drosophila. We have generated a TrxR2 transgenic (T-tg) mouse which has ubiquitously heighted (two-fold) TrxR2 expression. We found that overexpression of TrxR2 leads to enhanced mitochondrial metabolism and increased resistance to mitochondrial oxidative damage in MEFs (data not shown). We also found that female T-tg mice showed a leaner trend and reduced food consumption, with improved glucose tolerance but no difference in insulin sensitivity. These mice showed a lower Oxygen consumption and CO2 production with lower energy expenditure in individual metabolic cages. We further tested their exercise capacity where T-tg mice had a similar performance to control mice. These results suggest that TrxR2 overexpression can lead to some beneficial metabolic changes that need to be further understood. (Acknowledgments to Nathan Shock Lifespan assessment Core for the help developing all these assays in mice).

